# Usefulness of PET/CT with ^18^F-FDG in Patients with Differentiated Thyroid Carcinoma after Radioiodine Therapy: An Italian Multicenter Study

**DOI:** 10.3390/diagnostics11071264

**Published:** 2021-07-14

**Authors:** Luca Filippi, Viviana Frantellizzi, Fabio Monari, Elisa Lodi Rizzini, Elena Tabacchi, Riccardo Pirisino, Andrea Marongiu, Susanna Nuvoli, Oreste Bagni, Giuseppe De Vincentis, Orazio Schillaci, Angela Spanu

**Affiliations:** 1 Nuclear Medicine Unit, “Santa Maria Goretti” Hospital, Via Antonio Canova, 04100 Latina, Italy; r.pirisino@ausl.latina.it (R.P.); o.bagni@ausl.latina.it (O.B.); 2 Department of Radiological Sciences, Oncology and Anatomical Pathology, Sapienza University of Rome, Viale Regina Elena 324, 00100 Rome, Italy; viviana.frantellizzi@uniroma1.it (V.F.); giuseppe.devincentis@uniroma1.it (G.D.V.); 3 Radiation Oncology Unit, IRCCS Azienda Ospedaliero-Universitaria Di Bologna, Via Massarenti 9, 40138 Bologna, Italy; fabio.monari2@unibo.it (F.M.); elisa.lodirizzini@aosp.bo.it (E.L.R.); 4 Nuclear Medicine Unit, IRCCS Azienda Ospedaliero-Universitaria di Bologna, Via Massarenti 9, 40138 Bologna, Italy; tabacchielena@libero.it; 5 Unit of Nuclear Medicine, Department of Medical, Surgical and Experimental Sciences, University of Sassari, Viale San Pietro 8, 07100 Sassari, Italy; m.and@live.it (A.M.); snuvoli@uniss.it (S.N.); aspanu@uniss.it (A.S.); 6 Department of Biomedicine and Prevention, University Tor Vergata, Viale Oxford 81, 00133 Rome, Italy; orazio.schillaci@uniroma2.it; 7 IRCCS Neuromed, 86077 Pozzilli, Italy

**Keywords:** ^18^F-FDG, positron emission computed tomography, differentiated thyroid carcinoma, precision medicine, oncology

## Abstract

Background: our aim was to assess the diagnostic performance and clinical impact of ^18^F-FDG PET/CT in patients with differentiated thyroid carcinoma (DTC), previously treated with surgery and radioiodine therapy (RAI). Methods: patients subjected to ^18^F-FDG PET/CT for suspected DTC recurrence in three Italian nuclear medicine units were evaluated. Two different clinical settings were identified: clinical setting 1 included patients (n = 40) that were enrolled according to the American Thyroid Association guidelines (i.e., negative ^131^1-WBS and Tg level > 10 ng/mL); and clinical setting 2, that encompassed subjects (n = 26) with serum Tg ≤ 10 ng/mL but morphological findings suspected of relapse. PET/CT’s impact was scored as significant if it provided an indication for surgery, or led to a novel therapeutic decision. Results: In total, 51/66 patients (77.3%) were ^18^F-FDG positive, while 15 (22.7%) were negative. PET/CT showed an overall sensitivity and specificity of 84.4% and 75%, respectively. Sensitivity was higher in clinical setting 1 (89.1%) as compared to clinical setting 2 (76.1%), although this difference was not statistically significant (*p* = 0.83). PET/CT influenced clinical management in 28 cases (42.4%), without a significant difference between the 2 groups of patients (*p* = 0.6). Conclusions: our preliminary data, although limited by the retrospective nature of the study and possible selection bias, suggest that ^18^F-FDG PET/CT may be utilized for the detection of DTC recurrence in different clinical settings, with a meaningful impact on clinical management.

## 1. Introduction

Differentiated thyroid carcinoma (DTC), including papillary thyroid carcinoma (PTC) and follicular thyroid carcinoma (FTC), represents the most frequent endocrine malignancy [1]. Its prognosis is generally good, as the overall rate of relapsing/persisting disease does not exceed 20% following up-front therapy, which consists of total thyroidectomy followed by ablative radioiodine therapy (RAI) [2].

Following thyroidectomy, RAI has represented the standard of care for all DTC patients for many years. More recently, the American Thyroid Association (ATA) guidelines have become more restricted, recommending the use of RAI as an adjuvant treatment only in high-risk patients [1].

Radioiodine whole body scan (^131^I-WBS) has a crucial role in subjects affected by DTC. In particular, post-treatment ^131^I-WBS, performed some days after the administration of the therapeutic dose of ^131^I, proved useful to disclose additional lesions not detected by conventional imaging methods, such as neck ultrasound (US) or chest X-ray (CXR) [3].

The follow-up of patients with DTC after surgery and RAI is mainly based on the periodical measurement of thyroglobulin levels (serum Tg) of L-thyroxine therapy, or under thyrotropin (TSH) stimulated conditions, neck US and ^131^I-WBS. In regards to ^131^I-WBS, its most relevant disadvantages are represented by the low resolution of planar scintigraphic images and the lack of anatomic localization, partially overcome by the introduction of hybrid SPECT/CT technology [4,5,6].

Positron emission computed tomography (PET/CT) with ^18^F-fluorodeoxyglucose (^18^F-FDG) is a well-established diagnostic approach in oncology, but presents a limited role in the initial phase of DTC due to its relatively low affinity for the aforementioned tracer [7,8]. However, during follow-up, some DTC patients treated with surgery plus RAI present with Tg levels that increase over time, with a negative ^131^I-WBS. This clinical condition is due to a dedifferentiation of DTC, in which the cells may lose their ability to incorporate ^131^I, while the glycolytic pathway is activated as the preferential source of energy for cell growth and proliferation [9]. In light of this, ATA guidelines suggest utilizing ^18^F-FDG PET/CT in DTC patients after surgery, with increasing serum Tg levels and negative ^131^I-WBS [9]. However, the clinical impact of ^18^F-FDG on the management of DTC in real-life clinical practice has yet to be fully addressed. In particular, current ATA guidelines recommend performing PET/CT with ^18^F-FDG in patients with Tg serum level > 10 ng/mL, although it has been reported that 10–20% of DTC patients with Tg inferior to the aforementioned threshold present PET positive findings [9].

In a recently published retrospective study on a large cohort (n = 173) of patients, Larg et al. found that ^18^F-FDG PET/CT resulted in 38% positive cases of DTC with increased Tg level and negative ^131^I-WBS [10]. It is noted that the authors report that 44% (n = 29) of the patients were subjected to surgery on the basis of ^18^F-FDG PET findings. It should underlined that although Tg represents an excellent biomarker for DTC follow-up, the presence of increased anti-Tg antibodies (anti-TgAb) can represent a major pitfall, leading to false negative results [11]. In such cases, the suspicion of DTC recurrence is mainly based on morphological imaging and ^131^I-WBS.

The aims of this retrospective study were: (1) to analyze the sensitivity of ^18^F-FDG PET/CT in DTC patients treated with at least one cycle of RAI with negative ^131^1-WBS and Tg level > 10 ng/mL *or* morphological findings (e.g., CT, CXR or US) suspected of relapse and serum Tg ≤ 10 ng/mL; (2) to determine PET/CT’s clinical impact on the therapeutic management of DTC patients included in the analysis.

## 2. Materials and Methods

### 2.1. Study Design

This multicenter retrospective observational Italian study was performed in 3 participating nuclear medicine units (S. Maria Goretti Hospital, Latina, University of Sassari, Sassari and S. Orsola-Malpighi Hospital Bologna). Firstly, all consecutive patients that were included were affected by DTC, treated with thyroidectomy and at least 1 cycle of RAI (i.e., dose ranged 1.1 GBq to 5.5 GBq/30–150 mCi) from March 2004 to December 2019, subjected to ^18^F-FDG PET/CT scan as a part of their diagnostic work-up.

Histopathological data, type of surgery, the results of performed diagnostic imaging (^131^I-WBS, neck US, CXR, ^18^F-FDG PET/CT) and laboratory tests (e.g., stimulated Tg and anti-Tg Ab levels) were recorded for each patient. Furthermore, the time that passed between DTC first diagnosis and the execution of PET/CT was registered. All the included patients had to present a complete and detailed available clinical history. Subjects with an increased value of anti-Tg Ab were excluded.

Subsequently, each involved nuclear medicine unit evaluated patients on a case-by-case basis and identified those who had undergone a PET/CT scan during follow-up among the initially enrolled subjects in one of the 2 following clinical conditions:

(a) clinical setting 1: negative ^131^I-WBS, last stimulated value of Tg, measured before PET/CT scan, >10 ng/mL, no other evidence of disease (neck and chest);
(b) clinical setting 2: negative ^131^I-WBS, morphological imaging (US, CXR or CT) suspected of relapse and last stimulated Tg value ≤10 ng/mL.

Patients who underwent a PET/CT scan in conditions that were different from the aforementioned clinical settings 1 or 2 (e.g., subjects presenting negative ^131^I-WBS, stimulated Tg > 10 ng/mL and also morphological imaging suspected of relapse) were excluded.

Clinical data of selected patients were collected in each center and then cumulatively gathered in an electronic database for analysis. The database, closed on January 2021, was analyzed between February 2021 and May 2021.

The primary endpoint of the study was to define the diagnostic performance of ^18^F-FDG PET/CT in all the enrolled patients. In particular, we evaluated whether PET/CT’s sensitivity for DTC recurrence detection was influenced by the time that passed between DTC diagnosis and PET/CT scan, or by the number of RAI cycles performed on the patients. Furthermore, PET/CT’s diagnostic impact in DTC patients belonging to the aforementioned clinical setting 1 and 2 was assessed.

The standard of reference to finally categorize PET/CT results (true-positive, true-negative, false-positive, false-negative) was histology when available, or, as an alternative, clinical and imaging follow-up data.

The secondary endpoint was to establish PET/CT’s clinical impact on change in DTC disease management. In each participating clinical center, patients’ follow-up was analyzed together with the referring physicians, who were asked to provide information on how clinical management was influenced by PET/CT results. In particular, PET/CT’s impact was scored as significant if: (1) it provided an indication for surgery of secondary lesions; (2) it entailed the implementation of a novel therapeutic regimen (e.g., tyrosine kinase inhibitor or external radiotherapy).

The management was considered to be adequate if the Tg serum level declined by more than 50% (compared to the baseline value) following treatment modification, or if the patients showed complete (CMR) or partial metabolic response (PMR), according to PET Response Criteria in Solid Tumors (PERCIST) at the follow-up PET/CT [12].

If more than one PET/CT scan was performed, only the first one was included for the analysis of sensitivity and clinical impact. PET/CT scans were defined as follow-up scans (i.e., FU Scans) if they were performed to evaluate treatment effect. Discrepancies were resolved through discussion among authors.

This retrospective study was approved by the local ethical committee of S. Orsola-Malpighi Hospital (prot. n. 1091/2016) and conducted in accordance with Helsinki Declaration of 1964 and later amendments. Major Italian nuclear medicine centers with experience in DTC management and PET/CT imaging were invited to participate. Informed consent was obtained from all individual participants included in the study before each PET/CT scan. No experimental procedures, novel devices or experimental drugs were used, and no funds were received.

### 2.2. ^18^F-FDG PET/CT

All the enrolled patients underwent PET/CT with ^18^F-FDG, according to present imaging guidelines [13], in conditions of stimulated TSH (through T4-wash-out or recombinant TSH administration). Each of the 3 centers involved in the study applied its own procedures and technology for PET/CT scan acquisition and image reconstruction. In brief, all patients fasted for at least 4 h before PET/CT scan and the blood glucose levels prior to tracer administration were set to <150 mg/dl. Whole body PET/CT scan was performed from the neck to the proximal tight between 60 and 70 min after the injection of 3.7 kBq/kg of ^18^F-FDG.

Images were interpreted by well-trained local readers. In particular, for each participating clinical center, a senior reader (i.e., more than 15 years of experience in PET/CT interpretation: L.F., S.N. and F.M.) reviewed PET images together with a junior reader (less than 15 years of experience: A.M., E.L.R. and R.P.). Any area with an uptake intensity greater than the background uptake that could not be identified as physiological activity (brain, heart, kidneys, bladder, urinary tract) was considered to be potentially pathological. For each patient, the sites and the number of sites with pathological uptake were annotated.

### 2.3. Statistical Analysis

Data are presented as mean ± standard deviation, median, or number (percentage). Statistical analysis was performed using a dedicated software (MedCalc 11.3.8.0; MedCalc Software, Mariakerke, Belgium). Sensitivity, specificity, positive and negative predictive values, and accuracy were calculated for ^18^F-FDG-PET/CT in all patients. Fisher’s exact test was applied to examine the differences in PET/CT’s sensitivity (primary endpoint) and change management (secondary endpoint) among groups, significance was established at 2-tailed *p* < 0.05. Retrospective analysis of sample size (i.e., margin of error and level of confidence calculation) was carried out through “Raosoft^®^ sample size calculator” (http://www.raosoft.com/samplesize.html (accessed on 7 March 2021)), on the basis of the epidemiologic data published by the Italian Association of Medical Oncology (https://www.aiom.it/wp-content/uploads/2020/10/2020_Numeri_Cancro-operatori_web.pdf (accessed on 7 March 2021)). This reporting an overall number of 13,200 expected new cases of thyroid cancer for the year 2020, assuming that 90% of these tumors are represented by DTC with a 20% rate of relapse after thyroidectomy and iodine therapy.

## 3. Results

**Figure 1:**
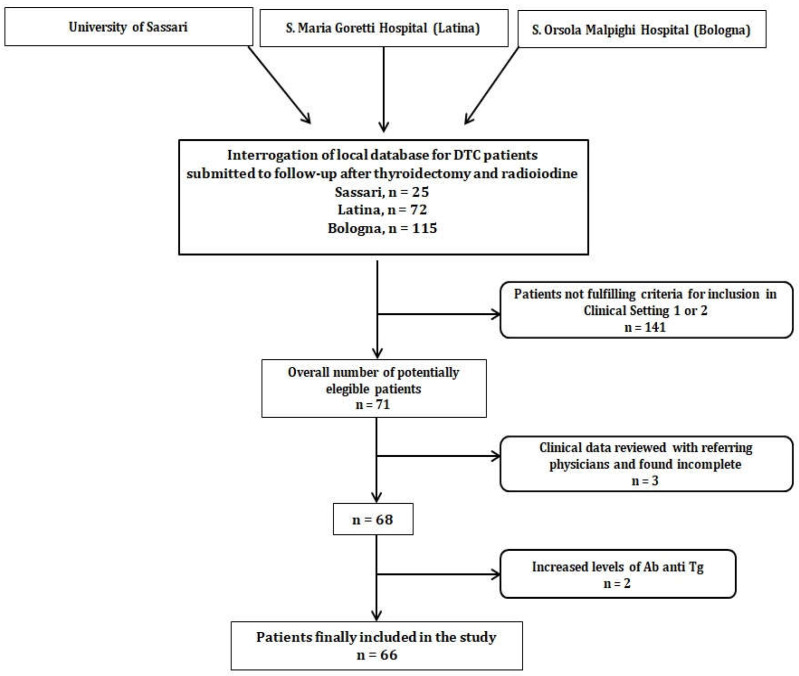
Diagnostic flow-chart of patient selection.

Every participating clinical center interrogated its own clinical database of DTC patients that attended follow-up after thyroidectomy and at least one RAI. An overall number of 212 subjects was identified. Subsequently, patients’ data were reviewed to check whether they fulfilled criteria for enrollment in clinical setting 1 or 2 (i.e., 71 subjects selected). After the exclusion of 3 patients due to increased anti-Tg Ab and 2 subjects with incomplete medical history, 66 patients were finally enrolled, as shown in Figure 1.

Clinical and demographic characteristics of the enrolled subjects are summarized in Table 1. In all subjects, thyroid carcinoma was classified according to the 8th edition of the AJCC/TNM staging system of thyroid cancer [14]. All were treated with at least 1 cycle of RAI, 5 out 66 (7.5%) patients received more than 1 cycle of RAI (range 2–4).

In regards to histology, 54 (81.9%) had PTC, including 11 cases of follicular variant and 4 cases of tall-cell variant, 8 (12.1%) presented FTC and 4 (6%) had other histotypes, including Hürthle cell carcinoma (n = 3) and adenoid-cystic carcinoma (n = 1).

According to ATA risk-stratification, 29 (44%) subjects were categorized as low risk, 18 (27.2%) as intermediate and 19 (28.8%) as high risk [1]. The median interval between diagnosis of DCT and the execution of ^18^F-FDG PET/CT scan was 17 months (mo) with an interquartile range (IQR) = 10.5–48 mo.

### 3.1. Diagnostic Performance of ^18^F-FDG PET/CT in Enrolled Patients

Among the enrolled patients, 51 (77.3%) presented ^18^F-FDG PET/CT positive findings, while 15 (22.7%) had negative PET/CT scans. Final diagnosis was established by histology (n = 22, i.e., 33.4%) or follow-up (n = 44, 66.6%). Of the 66 examined PET/CT scans, 49 were found to be true-positive, 6 true-negative, 2 false-positive and 9 false-negative. PET/CT showed good accuracy (83.3%) for the detection of DTC recurrence, with an overall sensitivity and specificity of 84.4% and 75%, respectively, while positive predictive value (PPV) and negative predictive value (NPV) resulted in 96% and 40.1%, respectively.

When patients who had received more than one cycle of RAI were excluded from analysis, PET/CT’s sensitivity resulted in 85.1%, not significantly different to that calculated in the overall enrolled population.

PET/CT’s sensitivity was further evaluated by stratifying patients into three different groups, according to the time passed between DTC first diagnosis and ^18^F-FDG PET/CT execution, by utilizing discrete intervals as follows: group 1 (n = 16, time from diagnosis ≤ to the 25th percentile, i.e., 10.5 mo), group 2 (n = 17, time from diagnosis between the 25th and 50th percentile, i.e., >10.5 and ≤17 mo), group 3 (n = 33, time from diagnosis > to the 50th percentile, i.e., 17 mo). PET/CT’s sensitivity resulted in 100%, 73.3% and 81.4% for the group 1, 2 and 3, respectively. No significant differences were found among groups (*p* = 0.83).

### 3.2. ^18^F-FDG PET/CT in Different Clinical Settings

Forty patients (60.6%) underwent ^18^F-FDG PET due to increased stimulated Tg level >10 ng/mL (mean Tg value 242.2 ± 560.7 ng/mL), negative ^131^I-WBS and no structural evidence of disease (clinical setting 1), while 26 (39.4%) with serum Tg value ≤10 ng/mL (mean value 1.8 ± 4.6 ng/mL) were submitted to PET/CT scan due to morphological findings (CXR = 4; US = 21; CT = 1) suspected of relapse (clinical setting 2).

The PET/CT’s sensitivity, specificity, PPV, NPV and accuracy resulted in 89.1%, 66.6%, 97%, 33.3% and 87.5%, respectively, for patients evaluated in clinical setting 1. For patients in clinical setting 2, sensitivity, specificity, PPV, NPV and accuracy were 76.1%, 80%, 94.1%, 44.5% and 76.9%, respectively. Through statistical analysis, no significant difference in PET/CT’s sensitivity was found between patients in clinical setting 1 and those in clinical setting 2 (*p* = 0.83). Table 2 summarizes PET/CT’s diagnostic performance in the overall population and in the different clinical settings.

**Figure 2:**
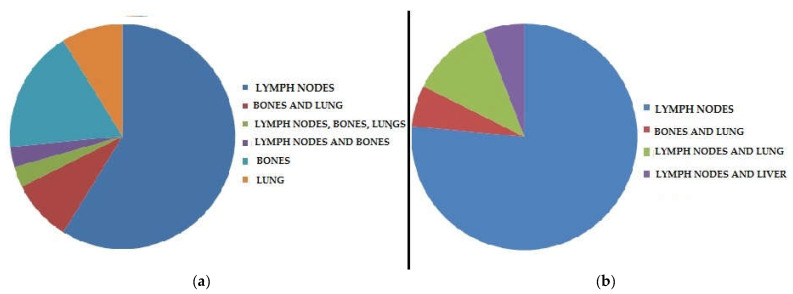
Graphic distribution of lesions detected by PET/CT in the 2 groups of patients: (**a**) patients (n = 40) examined in clinical setting 1; (**b**) patients (n = 26) evaluated in clinical setting 2.

The results of PET/CT, concerning the different sites of disease identified in each of the two clinical settings, are represented in Figure 2.

Among patients examined at clinical setting 2, PET/CT detected additional sites of disease in those identified as suspected by conventional imaging in 6 out of 17 patients (35%) with positive PET results, as detailed in Table 3.

### 3.3. Impact on Clinical Management

Change in management occurred in 28/66 patients (42.4%). In such cases, clinical decisions without considering PET/CT scans would have resulted in further cycle of RAI in 19 cases and strict follow-up in 9 subjects. The following changes occurred as a result of ^18^F-FDG PET/CT: 21 patients were submitted to surgery followed by an additional cycle of RAI, 6 patients received radiotherapy and 1 subject underwent systemic therapy with lenvatinib.

**Figure 3:**
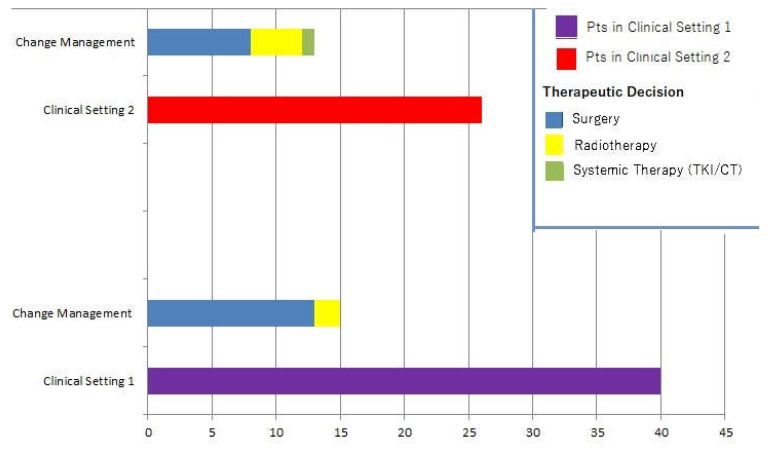
Illustration of PET/CT clinical impact on DTC patients. After PET/CT, subjects were switched to surgery, radiotherapy or systemic therapy.

PET/CT impacted 15 subjects belonging to clinical setting 1 (15/40, i.e., 37.5%) and 13 patients of the clinical setting 2 (13/26, i.e., 50%), respectively. No significant difference in PET/CT’s impact on clinical management was found between the two groups in the statistical analysis (*p* = 0.6). Figure 3 represents changes in therapeutic management.

**Figure 4:**
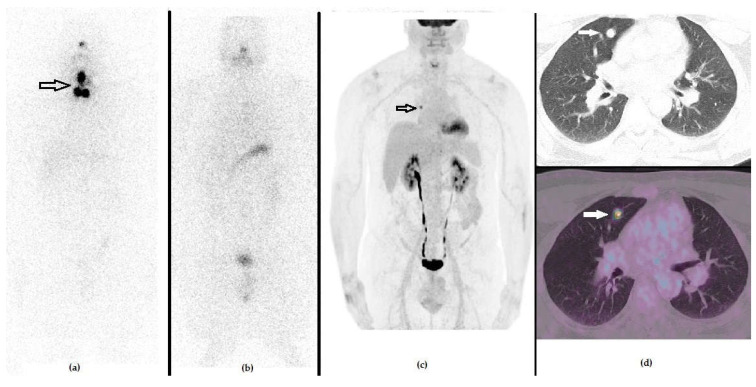
A 49-year-old male that previously underwent a thyroidectomy plus CND and 1 cycle of RAI due to PTC (pT3a N0 R0). The first panel represents the post-RAI ^131^I-WBS, showing the residual iodine-avid tissue in the anterior cervical region. (**a**) One year after RAI, he presented with an increased value of stimulated Tg serum level (i.e., 89 ng/mL), in spite of negative ^131^I-WBS (**b**) and no morphological signs of relapse at neck US or CXR. PET/CT with ^18^F-FDG demonstrated a focus of increased tracer uptake in the right lung, as evident by whole body (**c**) and axial CT (**d**, upper raw) and fused PET/CT ((**d**), lower raw) slices. The patient underwent surgery and histology was positive for PTC lung metastasis.

**Figure 5:**
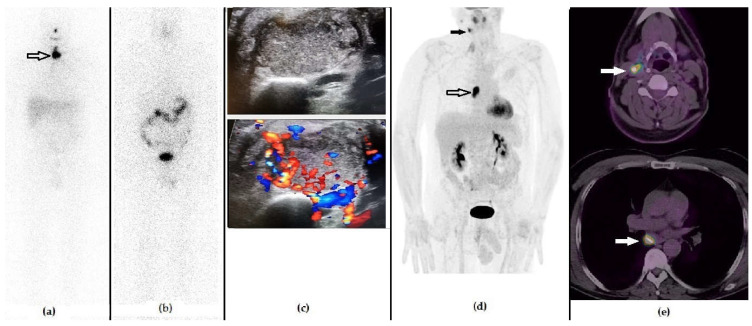
A 56-year-old male that previously underwent a thyroidectomy plus CND and 1 cycle of RAI due to Hürthle cell carcinoma (pT1b N1b). The first panel represents the post-RAI ^131^I-WBS showing the residual iodine-avid tissue in the anterior cervical region ((**a**), black arrow). Fifteen months after RAI, he presented with a slightly increased value of stimulated Tg serum level (i.e., 3.5 ng/mL), negative ^131^I-WBS (**b**) and neck US showing hypervascularized laterocervical node suspected of metastasis. (**c**) ^18^F-FDG PET/CT demonstrated a highly increased tracer incorporation in the suspected right laterocervical node, ((**d**) black arrow) also revealing metastases to the mediastinal nodes ((**d**), black bordered arrow), as evident in the whole body (**d**) and in the fused PET/CT axial images (**e**). The patient underwent surgery, followed by a further cycle of RAI.

Adequacy of changes to management was evaluated by PET/CT follow-up in 17 patients, of whom 10 presented CMR and 4 showed PMR, while biochemical parameters were utilized in the remaining 11 cases. PET-based therapeutic decision was considered adequate, according to the defined criteria in 85.7% (24/28) of the cases. Figure 4 and Figure 5 show emblematic clinical cases of DTC patients examined at clinical setting 1 and 2, respectively.

### 3.4. Statistical Analysis of Sample Size

Analysis of the selected sample size (n = 66) provided a margin of error equal to 9.62% with a confidence level of 69%.

## 4. Discussion

During DTC follow-up after surgery and RAI, some patients present with biochemical or structural signs of recurrence, in spite of a negative ^131^I-WBS, as a result of progressive DTC dedifferentiation. In such cases, PET/CT with ^18^F-FDG has been proposed for the detection of recurrence and therapy planning. However, the optimal criteria for identifying DTC patients to undergo a PET/CT scan remains a debated issue. According to ATA guidelines, PET/CT is recommended for subjects with negative ^131^I-WBS and stimulated Tg serum value >10 ng/mL [1].

In our retrospective study, we found that PET/CT can be useful not only for the detection of DTC recurrence in patients selected according to ATA criteria, but also in subjects with a low value of Tg serum and morphological imaging suspected of relapse. Of note, when our cohort was stratified in different groups according to the time interval between DTC diagnosis and PET/CT scan, we found the highest sensitivity (i.e., 100%) in subjects evaluated for suspected recurrence within 10.5 mo from diagnosis. This issue might be explained by a greater number of potentially non-specific or equivocal findings in subjects subjected to ^18^F-FDG PET/CT after a longer time interval from diagnosis.

Shammas et al. evaluated the clinical usefulness of PET/CT in 61 consecutive DTC patients, reporting a sensitivity and specificity of 68.4% and 82.4%, with a significantly higher rate of true positive findings for stimulated Tg serum level >10 ng/mL (60.8%), with Tg levels ranging 5–10 ng/mL (28.5%) or <5 ng/mL (22.2%) [15]. Of note, in the aforementioned research, PET/CT impacted patients’ clinical management in 27 out of 61 patients (44%), with surgery being the most common therapeutic decision.

Giovanella et al. investigated the relationship between Tg serum level and the results of ^18^F-FDG PET/CT in PTC patients with biochemical suspicion of recurrence [11]. The authors enrolled 42 patients with a regular follow-up after surgery and RAI, presenting with an increased Tg serum level, negative anti-Tg Ab and negative neck US/CXR/CT. All these subjects underwent a further cycle of RAI, and were then submitted to post-treatment ^131^I-WBS that failed to detect the sites of recurrence in all cases. Subsequently, the authors performed PET/CT with ^18^F-FDG that showed positive results in 29 out of 42 patients (69%). Sensitivity, specificity, NPV, PPV and accuracy of the ^18^FDG-PET/CT results were 93%, 84%, 93%, 84% and 90%, respectively. The optimal cut-off of stimulated Tg serum level for predicting positive PET/CT results resulted in 4.6 ng/mL, thus significantly lower than what is recommended by ATA guidelines (i.e., >10 ng/mL) [9].

Okzan et al. investigated the clinical usefulness of PET/CT in 61 patients affected by PTC and subjected to surgery and RAI, on suspicion of recurrence [16]. All the patients had negative ^131^I-WBS and 8 were also negative as shown by morphological imaging, while the remaining had uncertain findings at neck US or chest CT. Of note, in all cases, PET/CT results were compared with histology. At the final analysis, PET/CT’s sensitivity and specificity resulted in 80% and 27%, respectively. The authors further divided the cohort of enrolled subjects in two groups: one characterized by high Tg serum level, and another with high anti-Tg Ab levels. PET/CT’s sensitivity was higher in patients with increasing anti-Tg Ab levels than in subjects with elevated Tg (100 vs. 80%), but specificity remained low in both groups (30% and 27%, respectively). The authors hypothesized that the low value of specificity observed in their cohort might have been due to a high number of reactive neck lymph nodes, erroneously classified as metastatic by PET/CT.

Choi and coworkers investigated the role of ^18^F-FDG PET/CT after surgery and RAI in 84 patients with PTC, showing increased Tg serum levels or positive anti-Tg Ab. In regards to the first group, patients were further stratified according to the different Tg serum levels: in particular, in 39 patients with Tg levels between 2–10 ng/mL, the sensitivity, specificity, PPV, NPV and accuracy of ^18^F-FDG PET/CT were 50%, 97%, 50%, 97% and 97%, respectively [17].

Our results are in agreement with the previously cited papers [15,17], as we found that PET/CT was sensitive and specific for the detection of DTC recurrence. Worthy of note, similar to some of the aforementioned researches, we enrolled not only patients affected by PTC but also with other histologies, including tall-cell PTC variant and Hürthle cell carcinoma that are known to present a more aggressive biological behavior [18]. In regards to the subpopulation of patients characterized by low Tg serum level, our report showed that a detection rate slightly higher than that reported by Choi and colleagues [17]. This difference might be explained by the selection criteria, as in our group of patients belonging to the clinical setting 2, all subjects presented with suspicion of relapse during morphological imaging, aside from the relatively low value of Tg serum level.

A further consideration must be made concerning the impact of ^18^F-FDG PET/CT in the clinical management of patients with DTC recurrence. In this regard, Dennis et al. retrospectively analyzed 19 patients, that previously underwent RAI and ^131^I-WBS for PTC, with negative ^131^I-WBS and elevated serum markers (Tg or Ab anti-Tg) [19]. Of note, 11 out of the 15 patients with positive PET findings in the head/neck region were submitted to surgery or radiation therapy with the aim to remove all the ^18^F-FDG-avid sites. These 11 subjects had a median follow-up of 50 months, and 8 presented with marker levels below pre-scan values.

The results from Dennis’ group are substantially in line with those presented by Larg and colleagues [10], who found that PET/CT had a clinical impact in 89.2% (58/65) cases of DTC patients with increased Tg level and negative ^131^I-WBS. The majority of patients underwent surgery for removing the sites of abnormal ^18^F-FDG uptake. Of note, five subjects with positive PET findings were submitted to systemic therapy with tyrosine kinase inhibitors (TKI).

Our study confirms the significant impact of PET/CT in the management of patients with suspicion of DTC recurrence. In our series, in 6 of the 17 positive patients belonging to the clinical setting 2, PET/CT scan was able to identify additional sites of relapse in those categorized as “suspected” by morphological imaging. In particular, two subjects with altered neck lymph nodes at US were found to also present with pathological ^18^F-FDG uptake in mediastinal nodes. It is worth mentioning that the mediastinal node involvement represents an important prognostic factor in DTC patients [20], thus, its prompt and accurate detection significantly influences patient outcome.

In our cohort, one patient showing multiple ^18^F-FDG-positive skeletal and visceral metastases was switched to TKI. In agreement with the results from Larg et al. [10], PET/CT may be applied to identify DTC patients with advanced RAI-refractory diseased, who are more likely to benefit from TKI [21].

In regard to the study by Larg’s group, the authors analyzed PET/CT’s diagnostic impact in a group of 173 DTC patients evaluated after surgery and RAI, with increased Tg (i.e., >1 ng/mL) and negative ^131^I-WBS, reporting a PET/CT’s sensitivity of 88.09%, substantially in agreement with the value obtained in our cohort examined at clinical setting 1. Larg et al. found a significant difference in Tg levels among patients with positive and negative PET/CT scans. Of note, the authors did not include their analysis of patients with imaging signs (CT/US) of relapse, nor stratified PET/CT’s sensitivities by different intervals of Tg levels.

A methodological consideration must be made. Whether or not PET/CT with ^18^F-FDG in DTC should be performed under TSH stimulation remains a controversial issue. In our retrospective analysis, we included only patients who underwent PET/CT under TSH stimulation, as it has been reported that TSH stimulation not only influences PET/CT’s sensitivity, but also significantly affects its impact on clinical management [22,23].

Our study presents several limitations, such as its retrospective nature and the consequent potential for selection bias and the limited availability of histological correlation. Furthermore, the sample size included in our study is relatively small, although not significantly different to those in previously published papers on the same topic [11,15,16].

We did not consider any quantitative data in our analysis, such as maximum and mean standardized uptake values (SUVmax and SUVmean), as in this multicenter analysis different PET scanners and slightly different timing of acquisition were applied. In the future, the authors intend to perform a prospective, well designed study, in order to assess the value of PET/CT in a larger series, while also evaluating ^18^F-FDG PET-derived parameters, for the diagnosis and management of patients with suspected DTC recurrence. In order to determine a better Tg cut-off for identifying patients to undergo PET/CT, it would be desirable to select a population characterized by more homogeneous characteristics, especially in regard to the time interval between DTC diagnosis and PET/CT execution, ATA-risk class and histology. It might be hypothesized that more aggressive histologies, such as tall-cell variant and Hürthle cell carcinoma, might present during the dedifferentiation process as a reduced capacity to produce and secrete Tg, thus influencing the identification of a suitable cut-off value [24].

Despite the aforementioned limitations, we consider our results worthy of attention, taking into account the limited available data concerning the potential of PET/CT in clinical settings, other than those recommended by ATA guidelines. Although the cut-off of Tg level > 10 ng/mL may represent a useful tool for aiding clinicians to perform PET/CT in DTC patients with suspected relapse after surgery and RAI, it should be kept in mind that an ideal cut-off value of Tg is still far from being established. The patients’ overall clinical data, including the results of conventional imaging (US/CXR/CT), should be carefully considered to choose the most appropriate strategy and define personalized pathways of diagnosis and care.

## 5. Conclusions

In DTC patients’ follow-up after surgery and RAI, PET/CT with ^18^F-FDG may be recommended both in patients selected according to ATA guidelines and in subjects with a lower Tg serum level but suspected findings through morphological imaging. In our cohort, PET/CT impacted on the clinical management in 42.4% of the cases, providing useful information for PET-guided surgery or RT.

## Figures and Tables

**Table 1:** Summary of patients’ clinical demographic features.

Age (Years)		-
median	51	
mean ± SD	51 ± 19.2	
**Sex**	**n**	%
male	32	48.5
female	34	51.5
**Surgery**	**n**	**%**
Total thyroidectomy	37	56
Total thyroidectomy + CND	14	21.2
Total thyroidectomy + iLND	13	19.8
Total thyroidectomy + bLND	2	3
**Histology**	**n**	**%**
PTC	54	81.9
FTC	8	12.1
Others	4	6
**ATA Risk Stratification**	**n**	**%**
Low	29	44
Intermediate	18	27.2
High	19	28.8
**Previous RAI Therapies**	**Mean** ± **Sd**	**Range**
n of cycles	1.4 ± 0.6	1–4
**Clinical Setting before ^18^F-FDG PET/CT**	**n**	**%**
Clinical setting 1	40	60.6
Clinical setting 2	26	39.4
**Stimulated levels of Tg (ng/mL) before PET/CT**	**Mean** ± **Sd**	**Median**
All patients	144.8 ± 445.6	4.6
Clinical setting 1	242.2 ± 560.7	54.5
Clinical setting 2	1.8 ± 4.6	0.7

Abbreviations: CND—central neck dissection; iLND—ipsilateral neck dissection, bLND—bilateral neck dissection; PTC—papillary thyroid carcinoma, FTC—follicular thyroid carcinoma.

**Table 2:** Diagnostic performance of PET/CT in different clinical settings.

Clinical Setting	Positive PET/CTs	Se	Sp	Acc	PPV	NPV
Overall population	77.3%	84.4%	75%	83.3%	96.1%	40.1%
Clinical setting 1	85%	89.1%	66.6%	87.5%	97%	33.3%
Clinical setting 2	65.3%	76.1%	80%	76.9%	94.1%	44.5%

Abbreviations: Se—sensitivity; Sp—specificity; Acc—accuracy; PPV—predictive positive value; NPV—negative predictive value.

**Table 3:** Additional sites of disease detected by ^18^F-FDG PET/CT in patients with morphological findings suspected of relapse.

Patient	Morphological Site of Suspected Relapse	PET/CT Findings
1	bone (CT)	M+ (bone, lung)
2	Neck node (US)	Neck node, M+ (lung)
3	Neck node (US)	Neck and mediastinal nodes
4	Neck node (US)	Neck node, M+ (liver)
5	Neck node (US)	Neck node, M+ (lung)
6	Neck node (US)	Neck and mediastinal nodes

Abbreviations: US—ultrasound; CT—computed tomography; M+—visceral metastases.

## Data Availability

Datasets used or analyzed during the current study are available from the corresponding author on reasonable request.
